# Daily cost of delay to adequate antibiotic treatment among patients surviving a hospitalization with community-onset *Acinetobacter baumannii* pneumonia or sepsis

**DOI:** 10.1186/s13054-017-1719-9

**Published:** 2017-06-05

**Authors:** Marya D. Zilberberg, Brian H. Nathanson, Kate Sulham, Weihong Fan, Andrew F. Shorr

**Affiliations:** 1 EviMed Research Group, LLC, PO Box 303, Goshen, MA 01032 USA; 2 OptiStatim, LLC, Longmeadow, MA USA; 3 The Medicines Company, Parsippany, NJ USA; 4 Washington Hospital Center, 110 Irving St NW, Washington, DC 20010 USA

With >80% prevalence of multi-drug resistance, *Acinetobacter baumannii* (AB) poses a serious public health threat [1, 2]. We recently showed that inappropriate empiric therapy in the setting of community-onset AB pneumonia or sepsis is associated with 80% increase in hospital mortality [3]. The economic effects of delay in appropriate treatment are less clear. In a subgroup of the same cohort, we explored the cost associated with each day’s delay after obtaining index culture in instituting adequate therapy.

The original cohort derived from 176 US hospitals in the Premier Research database 2009–2013 and consisted of all adult patients admitted with pneumonia or sepsis as principal diagnosis, or as a secondary diagnosis in the setting of respiratory failure, along with antibiotic administration within 2 days of admission [3]. Only culture-confirmed infections were included. Inappropriate empiric therapy was present if the antibiotic administered did not cover the organism or if coverage did not start within 2 days of obtaining the positive culture. For the current analysis, patients were excluded if they did not survive the hospitalization or never in the hospitalization received adequate treatment (an agent that covered AB). “Day 0” to adequate therapy was the day the positive culture was obtained. To assign costs to delay in adequate treatment, we categorized length of stay (LOS) into three groups, as number of days: (1) until the first index culture (“pre” time); (2) after the index culture until the first appropriate antibiotic (period of interest); and (3) after the first appropriate antibiotic until hospital discharge (“post” time). We adjusted for pre and post times so that the costs associated with them were not attributed to the period of interest. The model structure was a generalized linear model (GLM) with a logarithmic link to account for the skew in total costs. In addition to the time variables, as in our prior study, covariates included other parameters known by hospital day 2 [3].

Of the 1423 patients in the original cohort, 460 (32.3%) were included in the current analysis. Among these, only 201 (43.7%) received appropriate therapy on day 0, with the median time to adequate treatment 3 days (interquartile range 1, 5). In the GLM, each day’s delay in instituting adequate therapy added $1344 (95% confidence interval $423, $2266, *p* = 0.004) to the total cost of hospitalization.

This analysis illustrates that delaying appropriate empiric treatment carries a financial cost that begins to accrue the moment infection is suspected and culture obtained. The fact that fully one-half of our population took ≥3 days to receive appropriate treatment, totaling > $4000/patient, equates to a substantial expenditure. Currently, in order to improve the likelihood of appropriate empiric coverage, and, in turn, the chances of survival, the only viable choice is to administer broad-spectrum therapy. However, since newer antibiotics with broader spectra are necessarily more expensive than older generic options, there is hesitation associated with the employment of newer alternatives. Our findings may refute the proposition that withholding novel agents represents a cost-minimization tactic.

## Abbreviations

AB: *Acinetobacter baumannii*
LOS: Length of stay
